# Optimizing the Number of Child Deaths Averted with Mass Azithromycin Distribution

**DOI:** 10.4269/ajtmh.19-0328

**Published:** 2020-02-17

**Authors:** Catherine E. Oldenburg, Ahmed M. Arzika, Ramatou Maliki, Ying Lin, Kieran S. O’Brien, Jeremy D. Keenan, Thomas M. Lietman, for the MORDOR Study Group

**Affiliations:** 1 Francis I. Proctor Foundation, University of California, San Francisco, San Francisco, California;; 2 Department of Ophthalmology, University of California, San Francisco, San Francisco, California;; 3 Department of Epidemiology and Biostatistics, University of California, San Francisco, San Francisco, California;; 4 The Carter Center Niger, Niamey, Niger

## Abstract

Biannual mass azithromycin distribution to children younger than 5 years has been shown to reduce all-cause mortality in sub-Saharan Africa. Antibiotic-sparing approaches to azithromycin distribution, such as targeting to younger children who are at higher risk of mortality, are being considered by policymakers. We evaluated the absolute number of deaths averted in the *Macrolides Oraux pour Réduire le Décès avec un Oeil sur la Résistance* study in three age-groups: 1–5 months, 1–11 months, and 1–59 months. The number of deaths averted decreased from 729 (95% CI 492 to 966) in children aged 1–59 months to 297 (95% CI 168 to 427) and 126 (95% CI 43 to 209) in the 1- to 11-month and 1- to 5-month groups, respectively. Limiting antibiotic treatment to a subgroup of higher risk children may result in fewer deaths averted compared with treating all preschool children.

## INTRODUCTION

Biannual mass azithromycin distribution has been shown to reduce all-cause postneonatal under-5 mortality in sub-Saharan Africa in the *Macrolides Oraux pour Réduire le Décès avec un Oeil sur la Résistance* (MORDOR) trial.^1^ In a prespecified subgroup analysis, the largest observed reduction in mortality was in children aged 1–5 months. Effect modification by age was not statistically significant. However, as the age-group with the greatest absolute risk of mortality,^2^ these children may have the most to gain from interventions that reduce child mortality. Targeting higher risk subgroups, such as younger age-groups or those in higher mortality geographic areas,^3^ may be tempting as it would limit antibiotic distribution, reducing the potential for resistance selection.^4^ However, if the ultimate goal is to avert the maximum number of deaths possible, mass distribution strategies may be more impactful. Here, we estimated the number of deaths averted in different age-groups compared with all 1- to 59-month-old children in the Niger site of MORDOR to understand the potential implications of an age-targeted program on the number of deaths averted with azithromycin distribution. The present analysis was restricted to the Niger MORDOR site because any implementation of azithromycin for the prevention of child mortality would most likely be restricted to geographic areas with under-5 child mortality rates more than 80 per 1,000 live births.

## METHODS

Complete methods for the MORDOR study have been reported (clinicaltrials.gov NCT02047981).^1^ Communities in the Boboye and Loga districts of Niger were eligible if they had a population of 200 to 2,000 inhabitants as per the most recent census. Eligible communities were randomized in a 1:1 fashion to 24 months of biannual mass azithromycin (20 mg per kg of body weight) or matching placebo distribution to all children aged 1–59 months in the community. Children living in these communities were eligible for treatment if they weighed at least 3,800 grams and were between one and 59 months of age. Treatment was administered during the census every 6 months. Ethical approval was obtained from the ethical committees of the Niger Ministry of Health and the Committee on Human Research at the University of California, San Francisco. Verbal informed consent was obtained from the guardian of all included children.

The primary, prespecified outcome was the community mortality rate as determined by the biannual census. A mortality event was counted if a child was recorded as alive and living in the household at the previous census and having died while living in the community at a subsequent census.

Analyses were restricted to the Niger site only, as any implementation of azithromycin for reduction in child mortality may be limited to settings with higher under-5 mortality.^5,6^ We calculated incidence rate differences for all-cause mortality in communities randomized to biannual azithromycin versus biannual placebo in children aged 1–5, 1–11, and 1–59 months at the time of treatment to estimate the absolute number of deaths averted per person-year based on the person-time observed in MORDOR-Niger. All analyses were conducted at the community level in R version 3.5.1 (the R Foundation for Statistical Computing, Vienna, Austria).

## RESULTS

**Figure 1.**
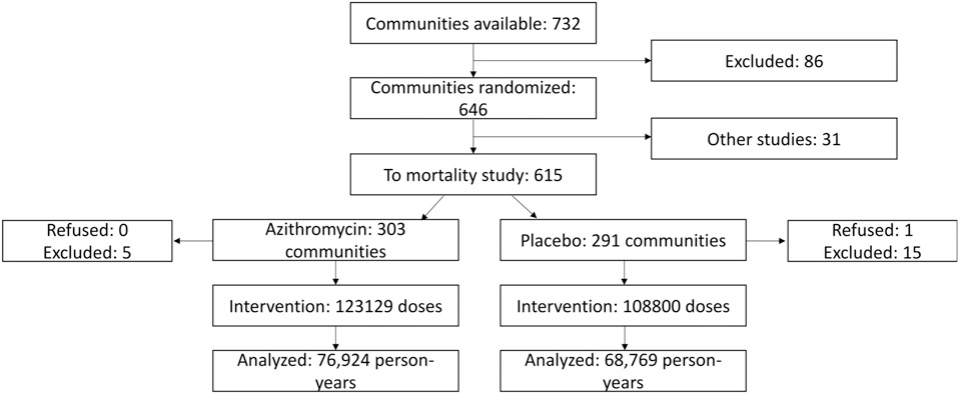
Consolidated standards of reporting trials flow diagram for the Niger site of the *Macrolides Oraux pour Réduire le Décès avec un Oeil sur la Résistance* study.

In 594 communities in Boboye and Loga, Niger, 3,615 deaths were observed over 145,694 person-years (Figure 1) from 2014 to 2017. Mortality in the placebo arm decreased with increasing age (Table 1), from 47.4 deaths per 1,000 person-years in children aged 1–5 months to 27.5 per 1,000 person-years overall. The incidence rate differences for azithromycin versus placebo were −5.0 (95% CI: −6.6 to −3.4), −11.5 (95% CI: −16.0 to −5.4), and −11.7 (95% CI: −19.4 to −3.9) deaths per 1,000 person-years in the 1- to 59-month, 1- to 11-month, and 1- to 5-month age-groups, respectively. This corresponded to 729 (95% CI: 492 to 966), 297 (95% CI: 168 to 427), and 126 (95% CI: 43 to 209) deaths averted in each age-group over the 24-month course of the trial.

**Table 1:** Number of deaths averted in children treated with azithromycin compared with placebo overall and by age-group, and Boboye and Loga districts, Niger

Age	Placebo			Azithromycin					
	Deaths	Person-years	Mortality rate per 1,000 py (95% CI)	Deaths	Person-years	Mortality rate per 1,000 py (95% CI)	Incidence rate difference per 1,000 py (95% CI)	Number needed to treat to avert 1 death (95% CI)	Number of deaths averted (95% CI)
1–5 m	244	5,152	47.4 (41.6 to 53.6)	202	5,657	35.7 (31.0 to 40.9)	−11.7 (−19.4 to −3.9)	85.5 (51.1 to 256.4)	126 (43 to 209)
1–11 m	584	12,299	47.5 (43.7 to 51.4)	488	13,563	36.0 (32.9 to 39.3)	−11.5 (−16.0 to −5.4)	87.0 (62.5 to 185.2)	297 (168 to 427)
1–59 m	1,888	68,769	27.5 (26.2 to 28.7)	1,727	76,924	22.5 (21.4 to 23.5)	−5.0 (−6.6 to −3.4)	200 (151.5 to 294.1)	729 (492 to 966)

m = month; py = person-years.

## DISCUSSION

The absolute number of deaths averted in the entire population was nearly 6-fold in the one- to 59-month-old population compared with limiting to only one- to 5-month-old children, and 2.5-fold higher than limiting to the one- to 11-month age-group. This suggests that a policy of targeted treatment would lead to fewer lives saved compared with mass treatment of all preschool-aged children. Although the efficacy (incidence rate difference) decreased as age increased, more total deaths were averted in the older age-groups because of the larger population size. The exact mechanism through which azithromycin prevents child mortality is unclear. A potential component could include untreated subsets of children benefiting indirectly from the treatment of other children via interruption of transmission of infection, similar to a “herd effect.”^7^ If true, including one- to 5-year-old children in distributions might help increase efficacy in the one- to 11-month-olds.

Including older children in treatment programs may not greatly increase program costs while maximizing the number of lives saved. Increasing the number of individuals treated in mass drug administration programs typically reduces the cost per treatment due to fixed delivery costs.^8^ A targeted program would still need to visit all communities and all households to ensure that all children in a given age range were treated. Treating additional older children would not add substantially to the cost of the program relative to treating only younger children, as major cost drivers include transport to the villages and house-to-house visits. Programs may not save substantial resources by limiting to the higher risk groups and would lose the opportunity to reduce mortality in lower risk groups. Including all preschool children in mass azithromycin distribution programs for child survival could maximize the number of deaths averted by these programs.

Limiting antibiotic distributions to specific targeted groups would reduce the number of antibiotic doses distributed. Mass azithromycin distribution for trachoma control has been shown to select for macrolide resistance in some potentially pathogenic organisms.^4,9,10^ Reducing the number of doses distributed and treating a smaller subset of the population would hypothetically lead to reduced selection for resistance relative to treating entire populations. Mathematical models suggest that the proportion of a population exposed to an antibiotic influences population-level resistance.^11,12^ Targeted programs would cover a smaller proportion of the population, potentially resulting in reduced prevalence of resistance. It is also unclear whether selection for resistance would affect treatment efficacy. Longer term results of the MORDOR trial suggested no waning effect of azithromycin for mortality prevention despite evidence of selection for macrolide resistance.^13,14^ Similarly, long-term use of cotrimoxazole prophylaxis in people living with HIV remains beneficial even in areas with high prevalence of cotrimoxazole resistance.^15^ Despite these reassurances, antibiotic resistance is an important concern with any antibiotic prophylactic program, especially if administered at a population level. Selection for resistance in important pathogenic organisms or indirect effects of resistance transmitted from children to other household members could be important consequences of azithromycin distribution for child survival. These problems might be mitigated to some extent by limiting distributions. Ongoing surveillance for resistance will be an important component of cost-benefit analyses weighing the advantages of potentially saving more lives by treating a larger subset of the population versus potentially reducing selection for resistance by treating fewer children.

These data must be considered in the context of several limitations. All preschool children regardless of age were treated as part of MORDOR. Estimates of deaths averted by subgroup, therefore, do not represent a scenario in which only that subgroup was treated. If there is any an indirect effect of azithromycin, the number of deaths averted in younger age-groups may be affected. The efficacy estimates presented here may be an overestimate of the true effect if there is a herd-like effect conferred by treating all children aged 1–59 months, and thus the difference in the absolute number of deaths averted between treating all children versus those aged 1–11 months could be even larger. No head-to-head comparison of targeting younger ages versus all preschool children has been conducted, so we are not able to comment on whether a targeted strategy would be as or more efficacious for reducing child mortality than treating all preschool children. As a non-prespecified secondary analysis of a randomized controlled trial, these results are hypothesis generating rather than conclusive and meant to provide insight into implementation and future studies of azithromycin distribution for prevention of child mortality.

Targeting higher risk groups is tempting, as it may reduce the amount of antibiotic distribution which could limit selection for resistance and may reduce some operating costs. However, this may constitute a “paradox of prevention”^16^—a far greater number of deaths may be prevented by treating an entire population than by targeting specific high-risk individuals.
